# Supplementary material for: HIVprotI: an integrated web based platform for prediction and design of HIV proteins inhibitors
Source: J Cheminform. 2018 Mar 9;10:12. doi: 10.1186/s13321-018-0266-y (PMC5845081; doi:10.1186/s13321-018-0266-y)
Supplement: Supplementary file 2 — Additional file 2. Source code of HIVProtI web server. [file 13321_2018_266_MOESM2_ESM.zip › HIVprotI_Source-code/hivprotI/weka-3-6-6/doc/weka/core/Randomizable.html]

Randomizable


---


|  |  |  |
| --- | --- | --- |
| **Overview** \| **Package** \| **Class** \| **Tree** \| **Deprecated** \| **Index** \| **Help** \| **Weka's home** | |  |
| **PREV CLASS**   **NEXT CLASS** | **FRAMES**    **NO FRAMES**     **All Classes** |  |
| SUMMARY: NESTED \| FIELD \| CONSTR \| METHOD | DETAIL: FIELD \| CONSTR \| METHOD |  |


---


## weka.core Interface Randomizable

**All Known Implementing Classes:**: AdaBoostM1, Agrawal, Bagging, BayesNet, BFTree, BIRCHCluster, ClassBalancedND, ClassificationGenerator, ClusterGenerator, Cobweb, CostSensitiveClassifier, CVParameterSelection, Dagging, DataGenerator, DataNearBalancedND, Decorate, EM, END, Expression, FarthestFirst, Grading, GridSearch, LED24, LogitBoost, MetaCost, MexicanHat, MiddleOutConstructor, MIEMDD, MultiBoostAB, MultiClassClassifier, MultilayerPerceptron, MultiScheme, ND, RacedIncrementalLogitBoost, RandomCommittee, RandomForest, RandomizableClassifier, RandomizableClusterer, RandomizableDensityBasedClusterer, RandomizableIteratedSingleClassifierEnhancer, RandomizableMultipleClassifiersCombiner, RandomizableSingleClassifierEnhancer, RandomizableSingleClustererEnhancer, RandomRBF, RandomSubSpace, RandomTree, RDG1, RegressionGenerator, RotationForest, sIB, SimpleCart, SimpleKMeans, Stacking, StackingC, SubspaceCluster, ThresholdSelector, Vote, XMeans

---

``` public interface Randomizable ```

Interface to something that has random behaviour that is able to be
seeded with an integer.

**Version:**
:   $Revision: 1.3 $

**Author:**
:   Richard Kirkby (rkirkby@cs.waikato.ac.nz)

---

| **Method Summary** | |
| --- | --- |
| `int` | `getSeed()`             Gets the seed for the random number generations |
| `void` | `setSeed(int seed)`             Set the seed for random number generation. |

**Method Detail**

### setSeed

```
void setSeed(int seed)
```

:   Set the seed for random number generation.

    :   **Parameters:**: `seed` - the seed

---


### getSeed

```
int getSeed()
```

:   Gets the seed for the random number generations

    :   **Returns:**: the seed for the random number generation


---


|  |  |  |
| --- | --- | --- |
| **Overview** \| **Package** \| **Class** \| **Tree** \| **Deprecated** \| **Index** \| **Help** \| **Weka's home** | |  |
| **PREV CLASS**   **NEXT CLASS** | **FRAMES**    **NO FRAMES**     **All Classes** |  |
| SUMMARY: NESTED \| FIELD \| CONSTR \| METHOD | DETAIL: FIELD \| CONSTR \| METHOD |  |


---
